# Supplementary material for: Complexome profiling of the Chlamydomonas psb28 mutant reveals TEF5 as an early PSII assembly factor
Source: Plant Cell. 2025 Mar 17;37(6):koaf055. doi: 10.1093/plcell/koaf055 (PMC12164590; doi:10.1093/plcell/koaf055)
Supplement: koaf055_Supplementary_Data [file koaf055_supplementary_data.zip › psb28_interactive.zip!/plots/intensity/qualityPlots/peptides/PSL3_Cre14.g617300.t1.2.html]

### Cre14.g617300.t1.2

Trivial name: PSL3  
SeqCoverage [median]: 14.00  
Mass: 64.32 kDa  
  
 

| Sequence | MissedCleavage | Mass [Da] | wtIntensity | muIntensity | Score |
| --- | --- | --- | --- | --- | --- |
| EQSLDGTNAGSVGGGGDGESAAAANR | 0 | 2347.03 | 0.00 | 1970.83 | 52.37 |
| GVSAAASGASALGAGR | 0 | 1301.67 | 2797.63 | 3406.38 | 182.23 |
| IADQEALQR | 0 | 1042.54 | 3437.66 | 5737.93 | 172.94 |
| LGASLVR | 0 | 714.44 | 2176.26 | 2419.84 | 86.51 |
| QVGGPLGEK | 0 | 883.48 | 2063.39 | 796.79 | 68.21 |
| VPVSEAVLYQR | 0 | 1259.69 | 845.09 | 7472.50 | 159.37 |
|  |  |  | 11320.0 | 21804.3 |  |
